# Longitudinally Extensive Transverse Myelitis in Autoimmune Connective Tissue Diseases: A Comprehensive Review

**DOI:** 10.7759/cureus.105862

**Published:** 2026-03-25

**Authors:** Guhan S R, Namasivayam K

**Affiliations:** 1 Department of General Medicine, K.A.P. Viswanatham Government Medical College, Trichy, IND

**Keywords:** autoimmune connective tissue diseases, immunopathogenesis, longitudinally extensive transverse myelitis, neuroimaging, prognosis

## Abstract

Longitudinally extensive transverse myelitis (LETM) is a severe inflammatory disorder of the spinal cord characterised by lesions extending across three or more vertebral segments and is associated with significant neurological morbidity. Autoimmune connective tissue diseases constitute an important noninfectious cause of this entity, reflecting complex immune-mediated and vascular mechanisms that result in extensive spinal cord injury. Despite increasing recognition, clinical presentation is heterogeneous, diagnostic differentiation remains challenging, and management strategies lack standardisation. The objective of this review is to synthesise current evidence on LETM occurring in the context of autoimmune connective tissue diseases, with emphasis on epidemiology, immunopathogenesis, clinical features, diagnostic evaluation, therapeutic approaches, and prognostic factors. A narrative literature search was conducted in PubMed/MEDLINE, Scopus, Web of Science, and Google Scholar covering studies published between 2000 and 2025 using predefined keywords related to LETM and autoimmune connective tissue diseases. Relevant studies were selected based on predefined thematic relevance and were narratively synthesised across clinical, radiological, laboratory, and therapeutic domains. The review highlights systemic lupus erythematosus as the most frequently associated disease, followed by Sjögren’s syndrome, systemic sclerosis, overlap syndromes, and antiphospholipid syndrome. Neuroimaging and serological evaluation play central roles in diagnosis, and early aggressive immunotherapy is consistently associated with improved neurological outcomes. LETM represents a rare but high-impact neurological manifestation of autoimmune connective tissue diseases. Improved awareness, timely diagnosis, and integrated multidisciplinary management are essential to optimise outcomes and reduce long-term disability.

## Introduction and background

Longitudinally extensive transverse myelitis (LETM) is a severe spinal cord inflammatory disease that is characterised by a continuous lesion stretching across three or more vertebral segments of the spinal cord on magnetic resonance imaging [1]. This radiological pattern is differentiated by short-segment myelitis and linked with a higher level of neurological morbidity, severe deterioration of functioning, and risk of permanent disability [2]. Clinical picture is generally characterised by acute or subacute weakness of the motor, sensory, and autonomous dysfunction, which develops to severe loss in the absence of timely treatment [3]. The etiological spectrum of LETM includes a wide array of infectious, neoplastic, metabolic, and immune-mediated etiologies of LETM, to which autoimmune mechanisms constitute a large proportion of noninfectious etiologies [4].

Autoimmune connective tissue diseases are a heterogeneous category of immune-mediated systemic disorders, which entail the presence of chronic inflammation, autoantibody formation, and multiple organ involvement [5]. Neurological manifestation forms an important but poorly known disease burden among this population [6]. The involvement of the central nervous system can consist of seizures, neuropsychiatric syndrome, cerebrovascular syndrome, and inflammatory myelopathies [7]. LETM is one of these manifestations, and often indicates increased systemic immune activation or extreme vascular or inflammatory pathology [8]. This widespread spinal cord involvement has been associated with disorders of systemic lupus erythematosus, Sjogren syndrome, systemic sclerosis, mixed connective tissue disease, and antiphospholipid syndrome with an unpredictable clinical course and prognosis [9].

LETM is the immunopathogenesis of an autoimmune connective tissue disease that is complex and multifactorial. Theories that have been put forward are immune complex deposition in the spinal cord vasculature, complement-mediated tissue damage, neuronal and glial damage by autoantibodies, inflammation by cytokines, and ischemic injury due to small-vessel vasculopathy or thrombosis [10]. Such pathways can be concomitant or interact with each other, causing extensive necrosis and inflammation of the spinal cord [11]. While several of these mechanisms are supported by evidence from specific connective tissue diseases, some components are inferred from general models of immune-mediated myelopathy. Neuroimmunology and imaging have improved the knowledge of these mechanisms, but there is still a high level of heterogeneity between various connective tissue diseases and even among individuals who have them [12]. This heterogeneity is a factor that leads to the differences in clinical manifestation, imaging features, and therapy response [2]. Diagnostic assessment is especially problematic. Connective tissue disease may have neurological symptoms that preclude systemic characteristics, which slow etiological assignment [13]. Radiological characteristics, being determined by the length of the lesion, tend to be similar to other types of inflammatory demyelinating diseases, such as neuromyelitis optica spectrum disorders and idiopathic transverse myelitis [14]. Serological findings, autoantibody patterns, and cerebrospinal fluid examination are valuable sources of diagnostic data, though their sensitivity and specificity to the different disease entities differ [15]. Lack of cohesive diagnostic algorithms often leads to misclassification or slow start of treatment and has a negative impact on the neurological outcomes [16].

The management of LETM with autoimmune connective tissue diseases is still not consistent [17]. The basis of acute therapy is composed of high-dose corticosteroids, which often are supplemented by plasma exchange or intravenous immunoglobulin in severe or recalcitrant cases [18]. Individualised long-term immunosuppressive or immunomodulatory therapy depends on the underlying connective tissue disease, degree of neurological involvement, and risk of relapse [19]. There are limited sources of evidence to assist in optimal therapeutic choices, treatment durations, and maintenance therapies, and most of the recommendations are based on observational studies and small case series [20]. The clinical prognosis is rather unpredictable, with significant neurological recovery and chronic disability being its extremes, which makes early diagnosis and vigorous treatment a crucial factor [21]. Following a growing interest in LETM as a severe neurological presentation of autoimmune connective tissue disease, existing data are disjointed and are mostly disease-specific [22]. There are few comparative studies done between the various subtypes of connective tissue disease, and no standardised method of diagnosis and management exists [23]. Future research on long-term neurological outcomes, predictors of relapse, and biomarker-mediated risk selection is scarce [24]. Where direct disease-specific evidence is limited, selected concepts in this review are interpreted in the context of broader inflammatory myelopathy literature and are identified accordingly.

Objectives of the review

This narrative review aims to synthesise current evidence on LETM associated with autoimmune connective tissue diseases, focusing on epidemiology, immunopathogenic mechanisms, clinical presentation, diagnostic strategies, and therapeutic approaches. The analysis seeks to identify knowledge gaps and clinically relevant insights to support improved recognition, risk stratification, and management of this severe neurological condition.

Methodology

A comprehensive literature search was performed using PubMed/MEDLINE, Scopus, Web of Science, and Google Scholar to identify studies addressing LETM associated with autoimmune connective tissue diseases. Studies published between 2000 and 2025 were included. Search terms consisted of combinations of "longitudinally extensive transverse myelitis", "autoimmune connective tissue diseases", "systemic lupus erythematosus", "Sjögren’s syndrome", "systemic sclerosis", "mixed connective tissue disease", and "antiphospholipid syndrome". Study selection followed a structured screening approach based on title, abstract, and full-text review for thematic relevance to connective tissue disease-associated LETM.

Studies were included if they reported clinical, radiological, laboratory, or therapeutic aspects of LETM in the context of autoimmune connective tissue diseases. Exclusion criteria comprised studies not addressing LETM, non-connective tissue disease etiologies, duplicate publications, and reports lacking clinical relevance. Selection was guided by relevance to the scope of this narrative review rather than a formal systematic framework. Studies that did not include autoimmune connective tissue diseases or articles that did not deal with LETM were excluded. Duplicate publications, lack of clinical specificity of editorial, conference abstracts, and reports were also excluded. Data from eligible studies were narratively synthesised based on thematic domains, including epidemiology, immunopathogenesis, clinical features, diagnostic evaluation, and therapeutic approaches.

## Review

Epidemiology and demographic patterns

LETM is an autoimmune connective tissue disease; using LETM as a disease marker is a rare but clinically important neurological manifestation [8]. The epidemiology is still limited as the incidence is low, different diagnostic criteria applied are heterogeneous, and retrospective cohorts and case series are used [1,17]. Existing evidence suggests that LETM is more frequent in various connective tissue diseases, which are characterised by various differences in immunopathogenesis, vascular involvement, and disease activity [13]. Systemic lupus erythematosus has the highest proportion of reported cases with its high prevalence, burden of systemic inflammatory and central nervous system involvement propensity [25]. Less common states, such as Sjogren syndrome and antiphospholipid syndrome, are less commonly related but show unequally severe neurological outcomes in the event of spinal cord involvement [26]. The patterns of the age distribution indicate that there were more individuals aged between young and middle-aged adults, with a majority being reported between the third and the fifth decades of life [17]. Children and geriatric presentations are rare and are normally reported as individual cases [1]. There is always a significant female preponderance whenever autoimmune connective tissue diseases are involved, which is analogous to the epidemiology of these pathologies and supports the role of sex-based immunological variation in susceptibility to diseases [27]. Contributory factors are hormonal effects, genetic susceptibility, and immune regulation [28].

Geographic patterns indicate a higher reporting rate in those regions that have already developed systems of autoimmune disease registries and accessibility to more developed neuroimaging systems [29]. The lack of representativeness of the low- and middle-income areas can be considered as a result of diagnostic shortcomings instead of the actual epidemiological disparities [6]. It has been proposed that there is ethnic variability, especially in LETM, which is associated with systemic lupus erythematosus, with a greater frequency of the latter presenting in populations of Asian and African ancestry, but there is little strong population-based evidence to support this claim [30].

The temporal patterns of the last decade show that there has been more appreciation of LETM in autoimmune connective tissue disorders, which is evidenced by the increase in magnetic resonance imaging resolution, expansion of autoantibodies, and clinical awareness [15]. One of the positive effects has been earlier diagnosis leading to a faster start of immunotherapy, but the total incidence has been shown to be stable, so this entity can be classified as a rare but high-impact one [1]. The epidemiological profile highlights the importance of increased alertness in high-risk groups, especially in young women who have active systemic autoimmune disease and show acute myelopathy symptoms [31]. Table 1 shows the most important trends in epidemiology and demographics of LETM among the major autoimmune connective tissue diseases.

**Table 1:** Longitudinally extensive transverse myelitis (LETM) demographics in autoimmune connective tissue diseases LETM: longitudinally extensive transverse myelitis, SLE: systemic lupus erythematosus, APS: antiphospholipid syndrome, MCTD: mixed connective tissue disease

Autoimmune connective tissue disease	Estimated frequency of LETM	Predominant age group	Sex predominance	References
Systemic lupus erythematosus (SLE)	Most common	20–40 years	Female	[31]
Sjögren’s syndrome	Uncommon	30–50 years	Female	[7]
Antiphospholipid syndrome (APS)	Rare	30–45 years	Female	[26]
Systemic sclerosis	Very rare	40–60 years	Female	[7]
Mixed connective tissue disease (MCTD)	Rare	25–45 years	Female	[9]

Immunopathogenesis of longitudinally extensive transverse myelitis in autoimmune connective tissue diseases

In autoimmune connective tissue disease-associated LETM, immunopathogenesis is thought to involve complex interactions of humoral and cellular immunity, with mechanisms supported by disease-specific studies (e.g., SLE, Sjögren’s syndrome, APS) and partly extrapolated from broader inflammatory myelitis literature [13,15]. Uncontrolled immune tolerance results in the persistent effect of the autoreactive lymphocytes and the generation of pathogenic autoantibodies that cause inflammatory damage in the central nervous system [15]. The spinal cord is the most susceptible to these immune processes because it has a rich vascular supply and is prone to microvascular immune-mediated destruction [32,33]. Autoantibodies are significant in the pathogenesis of connective tissue disease-associated LETM, particularly supported by evidence from systemic lupus erythematosus and Sjögren’s syndrome, with some mechanisms extrapolated from broader autoimmune myelitis literature [15]. The involvement of antinuclear antibodies, anti-double-stranded DNA antibodies, anti-Ro /SSA antibodies, and antiphospholipid antibodies in spinal cord injury has been linked to direct binding to neurons, development of immune complexes, and endothelial damage [34]. The deposition of immune complexes in the spinal cord vasculature stimulates complement activation, which causes a breakage in the blood-spinal cord barrier and recruitment of inflammatory cells [32]. Complement-mediated cytotoxicity increases the severity and progression of tissue injury and spreads the inflammatory lesions along longitudinal lines in several vertebral segments [33].

**Figure 1:**
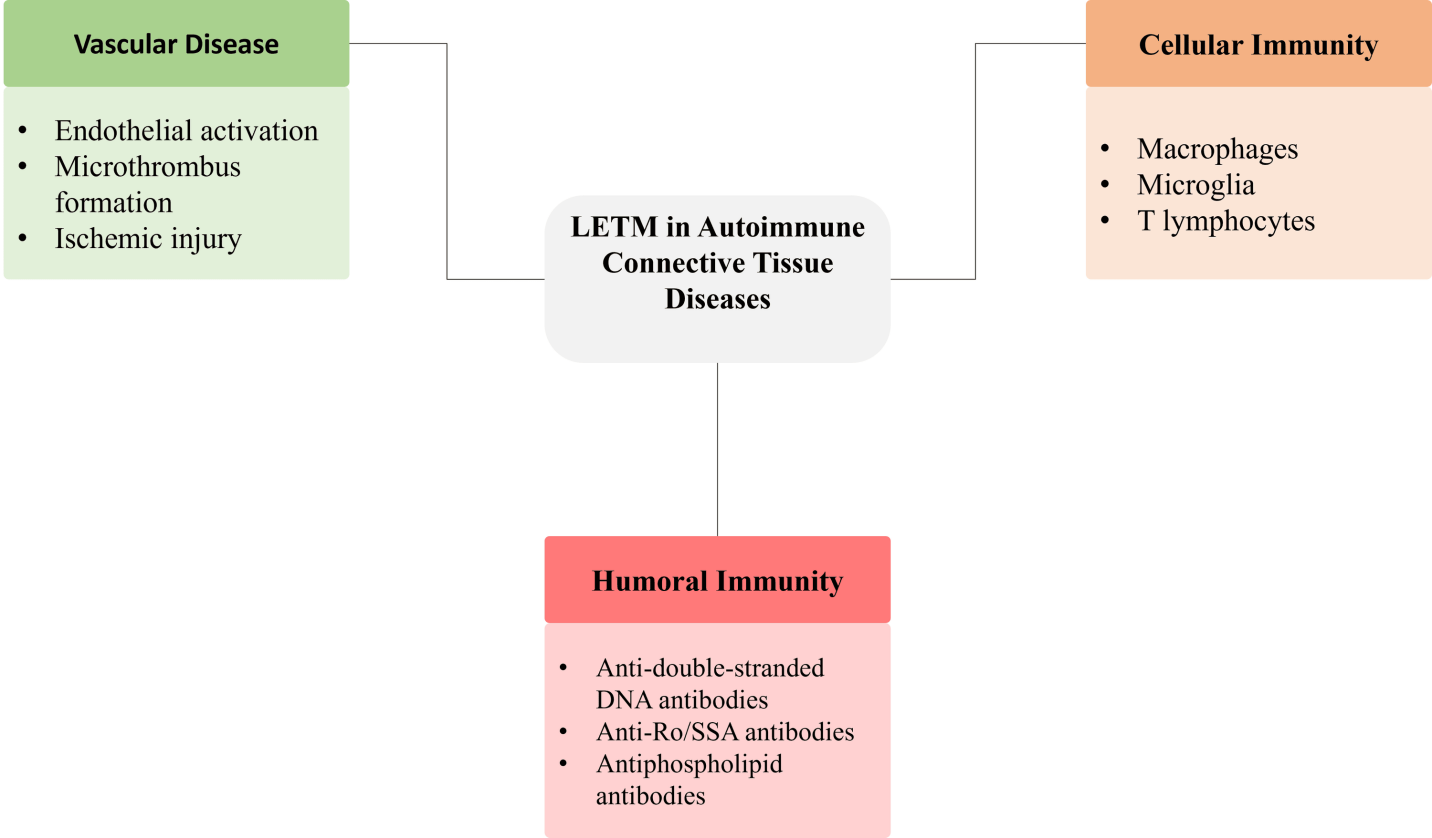
Immunopathogenesis of transverse myelitis in autoimmune connective tissue diseases LETM: longitudinally extensive transverse myelitis, anti-Ro/SSA: anti-Ro/Sjögren’s-syndrome-related antigen A, DNA: deoxyribonucleic acid, T lymphocytes: thymus-derived lymphocytes Image credit: Namasivayam K

There are also cellular immune responses that lead to disease progression [13]. The T lymphocytes activated penetrate the spinal cord parenchyma and discharge proinflammatory cytokines, including interleukin-6, tumour necrosis factor-α, and interferon-γ, which worsen the condition of local inflammation and stimulate demyelination [32]. The activation of macrophages and microglia enhances myelin degradation and axonal injury, leading to extensive neural dysfunction. B lymphocytes are also involved in the process of antigen presentation and the production of prolonged autoantibodies, which exacerbate the cascades of chronic inflammation [15]. Vascular mechanisms are particularly prominent in antiphospholipid syndrome-associated LETM, while similar processes may contribute to overlap connective tissue diseases [26]. Spinal cord perfusion is dysfunctional due to immune-mediated vasculopathy, endothelial activation, and microthrombus formation, resulting in ischemic injury on top of inflammatory injury [35]. The mutuality of immune-mediated inflammation and vascular damage is the cause of the severity, longitudinal course, and frequently rapid course of transverse myelitis in autoimmune connective tissue diseases [36]. Figure 1 shows the integrated immunopathogenic pathways of transverse myelitis, highlighting the roles of vascular mechanisms, humoral immunity, and cellular immune responses in autoimmune connective tissue diseases.

Systemic lupus erythematosus-associated longitudinally extensive transverse myelitis

LETM is most commonly linked to systemic lupus erythematosus, which is the autoimmune connective tissue disease [37]. The spinal cord involvement is frequent in the environment of active systemic disease, but in a smaller proportion of patients, neurological manifestations may appear before conventional systemic manifestations [38]. Clinical expression Clinical manifestation usually is acute or subacute, consisting of rapidly progressive paraparesis or quadriparesis, impairment of the sensory level, and autonomic dysfunction, such as that of the bladder and bowel [25,34]. Large lesion burden often leads to later devastating neurological impairment of its onset, which highlights the aggressive character of such manifestation [39]. Systemic lupus erythematosus-related LETM has a disease course that is not predictable, but may have monophasic disease with partial recovery, or relapsing or progressive disease with cumulative disability [36]. There is a frequent occurrence of early neurological degradation, especially when it is diagnosed too late or has a weak immunosuppression [34]. Longitudinally extensive T2 hyperintense lesions of the cervical or thoracic spinal cord, often with cord swelling and contrast enhancement, which represent active inflammation, are typical in magnetic resonance imaging [38]. There can be concomitant cerebral or optic pathway involvement, which makes it difficult to distinguish the clinical picture from other inflammatory myelopathies [40,41].

Immunology correlates are extremely important in the disease manifestation and prognosis [34]. Anti-double-stranded DNA antibodies of high titers and low levels of complement are often found with spinal cord involvement, which represent increased systemic immune activation [37]. The presence of antiphospholipid antibodies in a noteworthy percentage of affected individuals is linked to the damage of the vessels, microthrombotic, and worse neurological outcomes [26]. High levels of inflammatory indicators and pleocytosis of the cerebral fluid are additional evidence of immune-mediated pathogenesis [36]. The prognostic factors are the severity of the neurological deficit at presentation, the location of the spinal cord, and the presence of antiphospholipid antibodies and promptness in aggressive immunotherapy [26]. Promptness of high-dose corticosteroid therapy with immunosuppressive medication like cyclophosphamide has been linked to better functional outcome [36]. Continuous deficits and recurrence risk are high in those cases that have widespread cord necrosis or those that are not started early, thus the necessity of prompt management and early recognition [39]. Table 2 is the summary of the main clinical, radiological, and immunological features of LETM associated with systemic lupus erythematosus.

**Table 2:** Clinical, radiological, and immunological features of systemic lupus erythematosus-associated longitudinally extensive transverse myelitis LETM: longitudinally extensive transverse myelitis, SLE: systemic lupus erythematosus, MRI: magnetic resonance imaging, anti-dsDNA: anti-double-stranded deoxyribonucleic acid

Feature category	Common findings	Clinical significance	Prognostic implication	Supporting evidence	References
Clinical	Acute paraparesis, sensory level, and autonomic dysfunction	Rapid functional decline	Severe onset linked to poorer recovery	Observational studies	[37]
Radiological	Longitudinal T2 hyperintensity, cord swelling	Confirms LETM diagnosis	Greater lesion length predicts disability	MRI-based cohorts	[37]
Immunological	Anti-dsDNA positivity, low complement levels	Active immune disease	High titers linked to relapse	Serological analyses	[11]
Vascular	Antiphospholipid antibody positivity	Microvascular injury	Associated with worse outcomes	Clinical correlations	[26]
Therapeutic	Steroids, cyclophosphamide	Neurological improvement	Early treatment improves prognosis	Case series	[39]

Sjögren’s syndrome and longitudinally extensive transverse myelitis

Sjogren syndrome is a chronic autoimmune connective tissue disorder that is lymphocytic, in the exocrine glands, and with a broad range of general manifestations [5]. Neurological involvement is a severe extraglandular symptomatology and can involve the peripheral and central nervous system [13,23]. Although rare, the involvement of the spinal cord is one of the most serious neurological complications [7]. The LETM linked to Sjjoern syndrome is known to cause an acute or subacute myelopathy characterised by progressive motor deficit, sensory loss, and autonomic dysreflexia, which often leads to severe functional impairment [42]. LETM has been associated with both primary and secondary types of Sjögren syndrome. In other instances, myelitis is the initial manifestation of the classical sicca symptoms that result in the delay in diagnosis and under-acknowledgement of the underlying autoimmune condition [42]. This unusual relationship with time shows the significance of the consideration of Sjogren syndrome in a patient whose main manifestation is extensive inflammatory myelopathy of unidentified aetiology [1,4]. The presence of the spinal cord in Sjogren syndrome is more likely to undergo relapses as compared to other connective tissue diseases, and this leads to accumulated neurological deficit.

**Figure 2:**
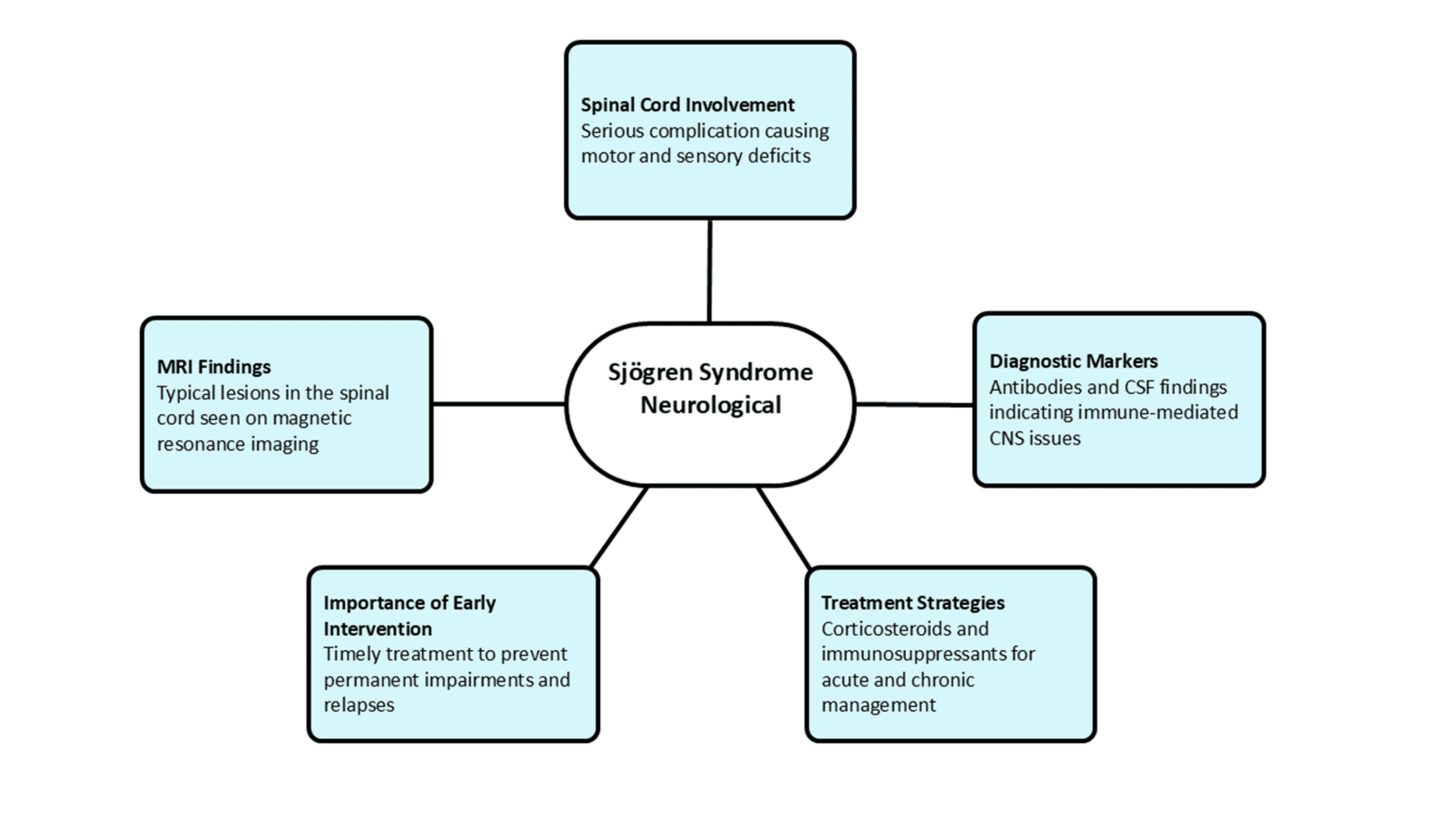
Neurological involvement in Sjögren syndrome: clinical features, diagnosis, and management MRI: magnetic resonance imaging, CSF: cerebrospinal fluid, CNS: central nervous system Image credit: Namasivayam K

There are unique immunological characteristics that help in diagnostic assessment [15]. These are anti-Ro/SSA and anti-La/SSB antibodies that are commonly observed and are the major indicators of immune-mediated central nervous system involvement [42]. The examination of cerebrospinal fluid usually shows the presence of lymphocytic pleocytosis and high levels of protein that prove an inflammatory pathogenesis [4,3]. Longitudinally extensive T2 hyperintense lesions, usually of the cervical and thoracic spinal cord, with inconsistent contrast gain, are typical of magnetic resonance imaging [14]. Lesions can be patchy, central cord, or both, and sometimes can resemble neuromyelitis optica spectrum disorders [8]. Response to therapy is not uniform; high-dose corticosteroids are the basis of acute therapy [3]. Cyclophosphamide or rituximab are immunosuppressive agents that have demonstrated efficacy in cases of recurrence or refractory cases [42]. Early identification and timely immunotherapy do not cease to be the most important factors of the neurological outcome since the later the treatment, the more likely it is to have permanent impairments and a high risk of relapses. Figure 2 shows the key features of neurological involvement in Sjögren syndrome, including spinal cord involvement, MRI findings, diagnostic markers, early intervention, and treatment strategies.

Systemic sclerosis and overlap syndromes

Systemic sclerosis is a persistent autoimmune auto-aggregative connective tissue disorder, which deals with the dysregulation of immune responses, vasculopathy, and fibrosis, which progresses in the skin and internal organs [43]. The presence of neurological involvement is rare in comparison with other connective tissue diseases, but the pharmaceutical implications of clinical manifestations of central nervous system involvement are significant in any case [13]. The spinal involvement, such as LETM, is uncommon but linked with the loss of the worst neurological functions and a poor functional outcome [44]. There are reported cases of acute or subacute onset of motor weakness, sensory disturbances, and autonomic dysfunction, most commonly presenting in patients with an established systemic autoimmune disease such as Neuromyelitis Optica Spectrum Disorder [45]. LETM associated with systemic sclerosis is believed to have a strong relationship with vascular damage and immune-mediated inflammation pathophysiology [32]. Endothelial dysfunction, chronic microangiopathy, and deposition of immune complexes all help to undermine the perfusion of the spinal cord and secondary inflammatory damage [32]. This is a vascular-dominant process that distinguishes the myelitis of systemic sclerosis and demonstrates the mainly antibody-mediated processes of other connective tissue diseases [15]. Longitudinally extensive T2 hyperintense lesions are usually seen through magnetic resonance imaging, sometimes with evidence of ischemic damage being present, as a result of both inflammatory and vasculopathic pathways [2].

Additional diagnostic issues are relevant to overlap syndromes that are characterised by the presence of clinical and serological phenomena of several connective tissue diseases [46]. Other conditions can involve the spinal cord with a combined immunological signature, e.g., mixed connective tissue disease or overlap of systemic sclerosis and systemic lupus erythematosus [41]. In such cases, attribution of LETM to one disease entity is commonly challenging, and this makes it difficult to make therapeutic decisions and to determine the prognosis [40]. Autoantibody profiles most often show mixed results, such as anti-U1 ribonucleoprotein antibodies and antinuclear antibodies, making the etiological classification more difficult [9]. Treatment interventions are still highly experimental and based on the autoimmune myelopathies that are related [19]. Corticosteroids are usually used in high-dose therapy, especially when it is in the acute phase, and the adjunctive immunosuppressive therapy is thought to be effective in the severe or progressive stages [45]. Spinal cord involvement in systemic sclerosis and overlap syndromes is a condition that needs to be identified early because late diagnosis is linked to impairment of the condition and irreversible neurological deficits [35].

Antiphospholipid syndrome and vascular mechanisms

Antiphospholipid syndrome is an autoimmune disease characterised by frequent thrombotic incidents and adverse consequences of pregnancy in connection with ongoing antiphospholipid antibodies [26]. Neurological is also typical and occur as a result of vascular pathology, both of the large and the small vessels [35]. Spinal cord involvement is a rare but serious complication, which has given rise to LETM as a unique and clinically aggressive manifestation [1]. The development of neurological deficits is acute and is characterised by motor weakness, sensory loss, and autonomic dysfunction, and in many cases, the deficits are severely disabling [3,14]. The causal mechanism of LETM in antiphospholipid syndrome is more of a vascular than an inflammatory nature [26]. The antiphospholipid antibodies stimulate endothelial activation, platelet aggregation, and the breakdown of anticoagulant pathways, which cause microvascular thrombosis and spinal cord ischemia. Hypoperfusion of the spinal cord results in widespread tissue damage across multiple vertebral segments, explaining the longitudinal nature that is visible in neuroimaging [8]. This can co-exist with inflammatory processes, especially in patients with secondary antiphospholipid syndrome related to systemic lupus erythematosus, which compounds the damage to the spinal cord [25].

Longitudinally extensive T2 hyperintense lesions, often centred on the thoracic cord of the spinal cord, and sometimes with diffusion restriction, indicative of ischemic injury, are often seen on magnetic resonance imaging [14]. Patterns of contrast enhancement can be less intense than in mainly inflammatory myelopathies; that is, the ischemic aspect of tissue injury [2]. Findings of cerebrospinal fluid are uncharacteristically non-specific, or they are borderline inflammatory, with a vascular-dominant pathogenesis [4]. Management Therapeutic management needs to be combined to cover both immune-mediated and thrombotic processes [1]. Anticoagulation is a crucial element of therapy to avoid persistent or repetitive vascular damage [26]. Corticosteroids of high dose or immunosuppressants are not uncommonly used in cases of overlapping inflammatory appearances or a combination of connective tissue disease activity [35]. The timeliness of diagnosis and anticoagulant initiation is closely linked to prognosis, with delayed treatment being linked to the occurrence of irretrievable spinal cord infarction and long-term neurological impairments [2].

Radiological characteristics and neuroimaging patterns

Neuroimaging has been used to focus on the diagnosis and etiological discrimination of LETM in relation to autoimmune connective tissue diseases [47]. Spinal cord magnetic resonance imaging is the most suitable imaging modality, which is sensitive in identifying the extent and location of the lesion as well as the inflammatory alterations [14]. Continuous lesions involving three or more vertebral levels on sagittal T2-weighted images characterise longitudinally extensive lesions, which involve extensive inflammatory or ischemic damage [8]. The lesions of the cervical and thoracic cord occur most commonly in autoimmune connective tissue diseases and tend to extend into the central grey matter and adjacent white matter tracts [35]. These patterns are most frequently reported in SLE- and Sjögren’s syndrome-associated LETM, although similar findings are described in other inflammatory myelopathies. Various imaging characteristics reported in connective tissue disease-associated LETM may assist in differentiation, although many features overlap with other inflammatory myelopathies [48]. In autoimmune connective tissue diseases, the spinal cord lesions typically show central or patchy T2 hyperintensity along with variable cord swelling, which is the manifestation of active inflammation and oedema [49]. Patterns of contrast enhancement vary and may be diffuse, focal, or peripheral enhancement, which is associated with the activity of the disease and the breakdown of blood-spinal cord barriers [50]. In contrast to neuromyelitis optica spectrum disorders, lesions in connective tissue diseases do not always implicate the area postrema and may not always have extensive lesions of the optic nerves [14]. The paraspinal soft tissue abnormalities and meningeal enhancement are generally absent compared with infectious myelitis [51].

The diagnostic accuracy is also improved by the use of advanced imaging techniques [2]. Diffusion-weighted imaging helps in the separation of inflammatory myelitis and ischemic cord injury, especially in antiphospholipid syndrome and vasculopathic overlap of the same [14]. The mapping of the apparent diffusion coefficient can indicate limited diffusion in acute ischemic lesions, which can justify a vascular process [35]. Gadolinium-enhanced sequences can give an understanding of the activity of inflammation and response to treatment, and the resolution of enhancement is in line with clinical improvement [50]. On follow-up imaging, spinal cord atrophy is a sign of irreparable tissue damage and unfavourable neurological outcome [52]. Brain imaging also takes part in the overall evaluation [53]. White matter lesions, small-vessel ischemic dysplasia or co-occurring optic pathway abnormalities can indicate systemic autoimmune response or overlap syndromes [54]. A combination of the spinal and cerebral imaging results enhances etiological attribution and informs the doctor in making decisions on immunotherapy [22]. Table 3 provides the major radiological peculiarities and clinical significance of LETM related to autoimmune connective tissue diseases.

**Table 3:** Neuroimaging characteristics of longitudinally extensive transverse myelitis in autoimmune connective tissue diseases LETM: longitudinally extensive transverse myelitis, MRI: magnetic resonance imaging, SLE: systemic lupus erythematosus, APS: antiphospholipid syndrome

Imaging feature	Typical findings	Diagnostic value	Associated disease pattern	Prognostic implication	References
T2-weighted MRI	Longitudinal hyperintensity ≥3 vertebral segments	Confirms LETM	SLE, Sjögren’s syndrome	Longer lesions linked to disability	[46]
Contrast enhancement	Patchy or diffuse enhancement	Indicates active inflammation	Active autoimmune disease	Enhancement resolution predicts recovery	[27]
Diffusion-weighted imaging	Restricted diffusion in select cases	Differentiates ischemia	APS, vasculopathy	Suggests a poorer outcome	[49]
Cord swelling/atrophy	Acute swelling, chronic atrophy	Disease staging	Chronic or relapsing disease	Atrophy predicts permanent deficit	[52]
Brain MRI findings	White matter or ischemic lesions	Supports systemic involvement	Overlap syndromes	Indicates higher relapse risk	[40]

Laboratory and serological evaluation

Serological assessment and laboratory analysis are a vital part of the evaluation of LETM in relation to autoimmune connective tissue disorders. These studies help to make etiological attribution, measure disease activity, and plan therapy [1,7]. Profiling of autoantibodies is useful in connective tissue disease-associated LETM, particularly where disease-specific antibodies (e.g., anti-dsDNA, anti-Ro/SSA, antiphospholipid antibodies) provide direct evidence, although some diagnostic principles are extrapolated from broader autoimmune neurology literature [9]. ANA antibodies are commonly found throughout the autoimmune connective tissue diseases, which is a general label of immune disorders [5]. Disease-specific autoantibodies, including anti- double-stranded DNA, anti-Ro/SSA, anti-La/SSB, anti-U1 ribonucleoprotein, and antiphospholipid antibodies, assist in the classification of disease diagnosis as well as risk classification [15]. Inflammatory markers add some background on global immune responses. During acute stages of the disease, a high erythrocyte sedimentation rate and high C-reactive protein levels are usually observed, but normal values do not rule out inflammatory myelopathy [55]. Immune complex-mediated activity is indicated by complement consumption, especially low C3 and C4 levels, which are more often than not related to the spinal cord pathology in systemic lupus erythematosus [31]. They are useful parameters (although not very specific to central nervous system pathology) in monitoring disease activity and response to therapy [37].

**Figure 3:**
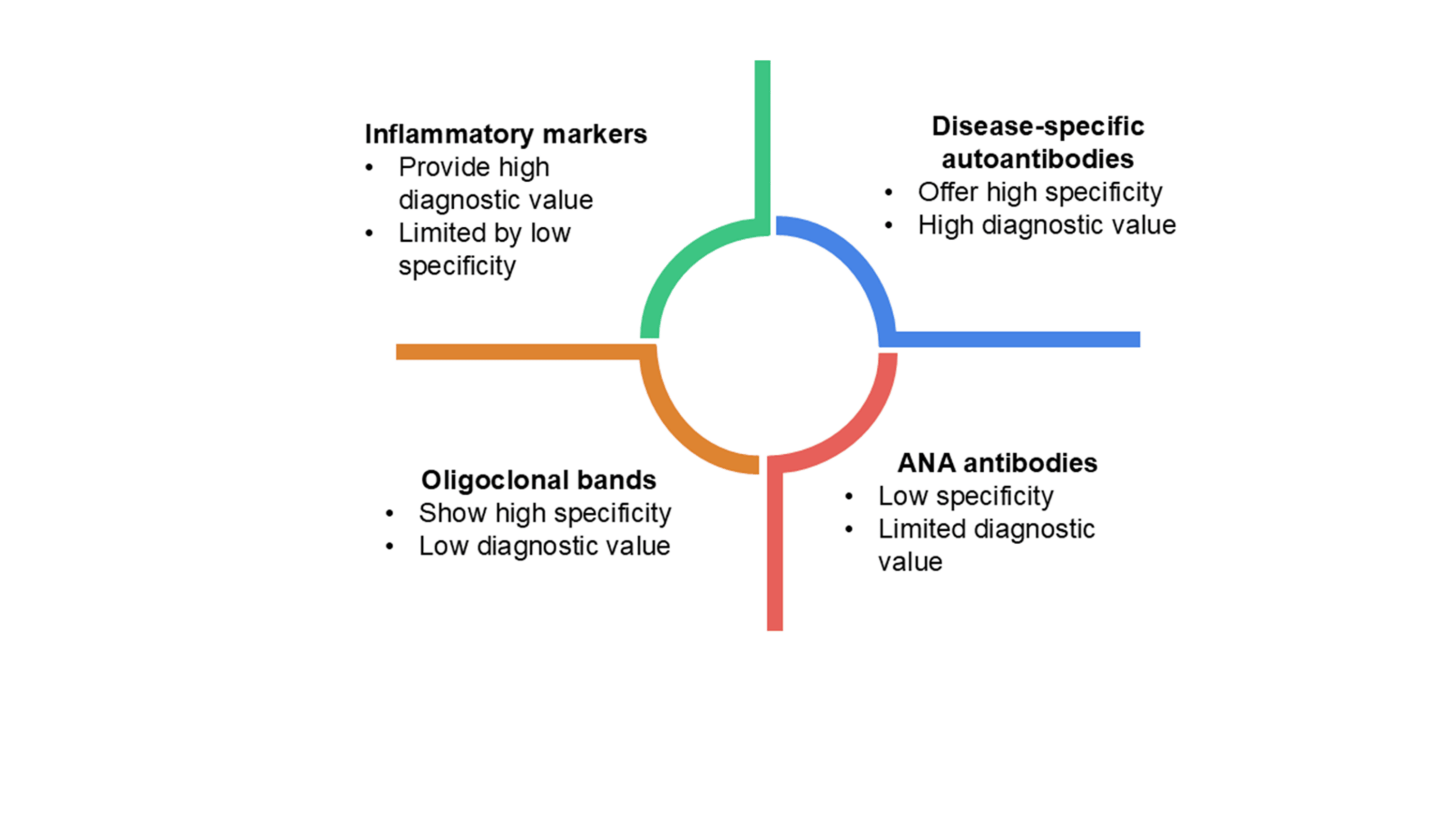
Diagnostic relevance of laboratory investigations in transverse myelitis ANA: antinuclear antibodies Image credit: Namasivayam K

Direct information on intrathecal inflammation is provided by analysis of the cerebrospinal fluid. Typical results are lymphocytic pleocytosis, high protein level, and high level of immunoglobulin production, which are indicative of an inflammatory pathogenesis [4]. Oligoclonal bands can be seen, but they are not disease-specific, and they are less uniform compared to primary demyelinating disorders [56]. The level of glucose in cerebral fluid is usually normal, and this helps to rule out the infection etiologies [4]. Lack of significant inflammatory deposits does not rule out immune-mediated myelitis, especially in vascular-dominated conditions like antiphospholipid syndrome [26]. There are significant limitations with laboratory and serological results in spite of their diagnostic value. The presence of autoantibodies can be antecedent or post-neurological, making it difficult to identify when [15]. Sharing serological profiles among the connective tissue diseases diminishes diagnostic specificity, especially when there is an overlap syndrome [5]. A combination of laboratory data with both clinical presentation and neuroimaging of LETM is a critical diagnosis for effective management. Figure 3 shows the comparative diagnostic value of key laboratory tests in transverse myelitis, highlighting differences in specificity and overall diagnostic utility among inflammatory markers, disease-specific autoantibodies, oligoclonal bands, and ANA antibodies.

Therapeutic strategies and clinical outcomes

Management of connective tissue disease-associated LETM is largely based on treatment approaches used in inflammatory myelopathies, with disease-specific adaptations informed by conditions such as SLE, Sjögren’s syndrome, and APS [35]. High-dose intravenous corticosteroids are usually used as acute therapy and help decrease inflammatory oedema, stabilise the blood-spinal cord barrier, and prevent the further development of neurological impairments [40]. Early corticosteroid treatment is linked to better outcomes in terms of function, as well as less lesion growth on neuroimaging [32]. Higher therapy levels are often necessitated in situations that portray poor clinical response to contain the disease [39]. Plasma exchange is an effective second-line treatment, with reported benefit in connective tissue disease-associated LETM, particularly in SLE and Sjögren’s syndrome, although evidence is largely derived from observational studies and broader inflammatory myelitis literature. The elimination of circulating autoantibodies, immune complexes, and inflammatory mediators leads to the neurological improvement of a minority of patients [44]. It has been suggested that clinical benefit occurs in a variety of autoimmune connective tissue disorders, such as systemic lupus erythematosus and Sjogren syndrome, especially when it is initiated early in the disease course [28]. IVIG is another rescue treatment, with immunomodulatory effects by blocking Fc receptors and regulating cytokines [41].

To enhance remission and minimise chances of relapse, immunosuppressive agents are normally used. Cyclophosphamide has proved to be effective in severe or recurrent cases, especially in systemic lupus erythematosus-associated myelitis [25]. Maintenance therapy includes other applied agents like azathioprine, mycophenolate mofetil, and rituximab and is chosen based on the underlying disease activity, comorbidities, and tolerability. The goals of long-term immunomodulation are to deactivate the immune system and prevent the recurrence of spinal cord inflammation [34]. The clinical course is extremely heterogeneous and relies on the severity at the onset of the disease, lesion location, underlying autoimmune disease, and the degree of intervention in time [35]. The most common finding is a positive response with respect to the recovery of patients who undergo early aggressive immunotherapy, where there is partial to significant recovery of motor and sensory functions [39]. Late onset of treatment is met with lifelong disability, repeated disease, and atrophy of the spinal cord on follow-up imaging [32]. These results demonstrate the relevance of early diagnosis, personalised immunotherapy, and prolonged follow-up of the disease to maximise the neurological prognosis in longitudinal extensive transverse myelitis.

Prognosis, complications, and predictors of recovery

The prognosis of LETM in connection with autoimmune connective tissue diseases is extremely dynamic and directly proportional to the severity of the disease, the degree of spinal cord impairment, as well as immunopathological mechanisms [40]. The functional outcomes vary between almost complete neurological recovery and long-term motor, sensory, and autonomic impairments that need the assistance of long-term care [25]. Big lesions relating to critical components of the spinal cord are always linked to increased disability burden and low chances of complete functional rehabilitation [14]. Early neurological status can be considered as one of the best predictors of outcome. Acute motor weakness, absolute sensory deficit, and premature dysfunction of the sphincter during presentation are associated with reduced recovery and higher chances of a lasting disability [47]. Radiological conditions also affect prognosis, whereby the length of the lesion, cord swelling, and later atrophy of the spinal cord on the follow-up image are associated with poor prognosis conditions [2]. The existence of comorbid cerebral or optic pathway involvement is an additional risk factor that enhances the chances of recurrence and progressive neurological dysfunction [34].

Additional prognostic information can be gained using immunological and vascular factors. The markers of high disease activity, continued positivity of autoantibodies, and complement consumption are linked to continued immune-mediated destruction and relapse propensity [15]. The presence of antiphospholipid antibody is associated with ischemic complications, frequent myelopathic exacerbation, and impaired neurological recovery [26]. Lateness of immunotherapy or insufficient suppression of the disease predisposes to the irreversible damage of axons and permanent disability [19]. The LETM complications are associated with severe effects on the quality of life. Most frequently reported sequelae include chronic motor impairment, neuropathic pain, spasticity, dysfunction of the bladder and bowel, and sexual dysfunction [13]. Recurrent infection, pressure injuries, and thromboembolism are also causes of secondary complications that contribute to morbidity [25]. Patients with long-term deficits often experience psychological distress and lack functional independence, which highlights the importance of providing long-term care [7]. The recovery paths are best in scenarios that have an early aggressive immunotherapy, reduced lesion load, and satisfactory long-term disease control [37]. Multidisciplinary rehabilitation and prolonged immunomodulatory therapy are important in achieving optimal functional recovery as well as reducing disability progression [1]. Detection of high-risk features enables tailored treatment and prognostic advice in the impacted case [35].

Limitations and future recommendations

Existing evidence on LETM in autoimmune connective tissue diseases has the limitation of being largely based on retrospective research, small case series, and inconsistent diagnostic criteria, and the findings cannot be generalized. The differences in imaging protocols, serological tests, and treatment regimens limit the ability to directly compare studies and disease subtypes. Lack of standard outcome measures and varied follow-up periods are also a hindrance to proper long-term neurological recovery and risk of relapse evaluation.

Prospective, multicenter studies that use standardised imaging and laboratory protocols and uniform definitions of diagnosis should be the focus of future studies. Creation of disease-specific biomarkers and confirmed prognostic models can improve early risk stratification and therapeutic individualisation. Further comparative research on the effectiveness of immunosuppressive and anticoagulation in various subgroups of connective tissue diseases is justified in order to guide the evidence-based practice. Long-term functional and quality-of-life outcomes would be beneficial to incorporate and offer a more holistic perspective on disease impact and inform the optimal patient-centered care.

## Conclusions

LETM represents a rare yet devastating neurological manifestation of autoimmune connective tissue diseases, marked by extensive spinal cord involvement and substantial risk of long-term disability. Its occurrence reflects complex interactions between immune-mediated inflammation, autoantibody activity, and vascular pathology, varying across disease subtypes such as systemic lupus erythematosus, Sjögren’s syndrome, systemic sclerosis, and antiphospholipid syndrome. Clinical presentation is frequently severe, diagnostic evaluation remains challenging, and delays in etiological attribution are common, particularly when neurological features precede systemic disease manifestations. Advances in neuroimaging and serological testing have improved diagnostic accuracy, yet significant heterogeneity persists in therapeutic strategies and reported outcomes. Early recognition and prompt initiation of aggressive immunotherapy emerge as critical determinants of neurological recovery, with lesion extent, disease activity, and vascular involvement serving as key prognostic factors. Despite growing awareness, current evidence remains fragmented and largely derived from low-level studies, underscoring the need for standardised diagnostic frameworks and outcome measures. A comprehensive, multidisciplinary approach integrating clinical assessment, imaging, laboratory evaluation, and long-term disease control is essential to optimise outcomes and reduce the burden of this severe neurological complication.
